# Mr. Woodham's Observations on Mr. Larrey's Cases

**Published:** 1805-02-01

**Authors:** James Woodham

**Affiliations:** West Smithfield


### *To the Editors of the Medical and Physical Journal.*

GENTLEMEN,

IN the last Number of the Medical and Physical Journal, are some observations, or more properly cases, by a Mr. Larrey. As these cases inculate ideas, at variance, I believe, with those of the soundest and most enlightened practitioners of this country, I beg leave to offer a few remarks on them.

The observations, which are seven in number, comprize the following diseases: Ophthalmia, a discharge from the nose resembling gonorrhœa, vertigo, phthisis pulmonalis, dysentery, erysipelas, and last of all, pain and stiffness of the limbs, attended with fever and symptoms of mania. The patients who were the subjects of these diseases, had all, excepting one, been previously affected with gonorrhœa, to the suppression of which Mr. Larrey attributes their complaints. Impressed with this idea, he imitated the suppressed discharges, either by the insertion of the matter of gonorrhœa into the urethra, or the injection of a strong alkaline solution; and the new running produced by these means, was what he thinks principally contributed toward their cure.

The infidel Gibbon has somewhere remarked, in his immortal work, "The Decline and Fall of the Roman Empire," that a latent and involuntary scepticism adheres to the most pious minds. The same may be affirmed of the inquirers after truth in the science of medicine. They not only require that the facts they are called upon to believe should be well authenticated, but that they should not be taken up hastily or upon slight grounds, or be contrary to the uniform experience of men of sound judgment and correct observation. If Mr. Larrey's observations are not built on too weak a foundation, they evidently militate against general experience. For, admitting the absorption (for so I must understand the term suppression as he uses it) of the matter of gonorrhœa, it by no means follows that the diseases in question were a consequence. The premises do not justify the conclusion. They have no more to do with the immediate effects of the absorption of venereal virus than gout, scrofula, atrophy, jaundice, and a thousand other diseases, which, as Mr. Hunter observes, were by the older surgeons attributed to it. What we should expect, and what, if any thing, follow the introduction duction of the venereal virus into the system, are bubos, peculiar ulcerations of the mouth, nose, and fauces, blotches of the skin, and affections of the bones, periosteum, tendons, and ligaments. None of these however were present, the ulceration and caries of the nose being the effect of the nasal gonorrhœa excoriating and eroding the parts, and not the direct consequence of absorption. Mercury, it is true, was employed in that case, as well as in many others, and undoubtedly was the chief cause of their cure; but it does not prove they were venereal: It only proves, what we all know, that mercury will cure other diseased actions as well as the veneral. Beside, is not absorption in gonorrhœa a very disputable point? It rarely, I believe, if ever happens; for if it did, as few modern surgeons I presume give mercury in that disease, none of their patients would escape a lues. Can the diseases we are speaking of, then, be said to have arisen from absorption of the matter of gonorrhœa? In all probability they were mere accidental circumstances, totally unconnected with the gonorrhœas which had preceded them; or, if any connection be admitted, they must have been what Darwin would have termed affections of remote sympathy. On this supposition the artificially produced discharge might have afforded relief, in the same way as the re-appearance of gonorrhœa in the contiguous sympathy of hernia humoralis removes the swelling of the epididymus or testis; yet granting this, it only comes in for a share of the cure, as in the majority of the cases (seven out of nine) mercury was had recourse to. The only fair cases are those of ophthalmia and phthisis; and on this last, as being of considerable importance, I shall hazard a few remarks. The case was that of a young lady, who was in the "third stage of a pulmonary consumption," and whose expectoration was "fetid, purulent, and greenish." She had previously laboured under leucorrhœa, which having been stopped by injections, gave birth, Mr. Larrey supposed, to her disease. By producing a purulent discharge from the vagina by means of a solution of volatile alkali, her most distressing symptoms began almost instantly to mitigate of their violence, and in a short time she perfectly recovered. Without being unreasonably sceptical, we may venture to say, that there must have been some fallacy in the case, and that it could not have been one of genuine phthisis pulmonalis. That a patient, in the third stage of pulmonary consumption, (that is, where the ulcerative process in the lungs had been some time going on) should recover at all, much less in a short period, is so contrary to what I may say every medical man is unfortunately in the habit of observing, that I fear even the most credulous would be disposed to doubt of such a fact. It is of no consequence, in my opinion, how an ulceration of the lungs originates, whether it be from tubercle or pneumonia, properly so called, whether it be from an idiopathic or a sympathetic affection; the effect is still the same, and we have the great difficulty to contend with, to heal an ulcer in so delicate a structure as the lungs, and which is *constantly* in contact with atmospheric air. Can we suppose therefore, can the utmost stretch of credulity believe, that simply a purulent discharge can, as it were by a miracle, effect a cure of that which has to this day continued, and will, in all probability, continue to be irremediable? My own experience, which has been pretty extensive, has convinced me, that when an ulcer of the lungs is once formed, no medicine, no not even the celebrated digitalis purpurea, or lichen islandicus, has a power of preventing those morbid actions going on, which sooner or later inevitably cut short the patient's life.

I am, &c.

*West Smithfield, Dec.* 14, 1804. JAMES WOODHAM.

---

